# Forgiveness Mediates the Relationship Between Middle Frontal Gyrus Volume and Clinical Symptoms in Adolescents

**DOI:** 10.3389/fnhum.2022.782893

**Published:** 2022-02-28

**Authors:** Eleanor M. Schuttenberg, Jennifer T. Sneider, David H. Rosmarin, Julia E. Cohen-Gilbert, Emily N. Oot, Anna M. Seraikas, Elena R. Stein, Arkadiy L. Maksimovskiy, Sion K. Harris, Marisa M. Silveri

**Affiliations:** ^1^Neurodevelopmental Laboratory on Addictions and Mental Health, McLean Hospital, Belmont, MA, United States; ^2^Department of Psychiatry and Harvard Medical School, Harvard University, Boston, MA, United States; ^3^Spirituality and Mental Health Program, McLean Hospital, Belmont, MA, United States; ^4^School of Medicine, Boston University, Boston, MA, United States; ^5^Department of Psychology, University of New Mexico, Albuquerque, NM, United States; ^6^Brain Imaging Center, McLean Hospital, Belmont, MA, United States; ^7^Center for Depression, Anxiety and Stress Research, McLean Hospital, Belmont, MA, United States; ^8^Center for Adolescent Behavioral Health Research, Division of Adolescent/Young Adult Medicine, Boston Children’s Hospital, Boston, MA, United States; ^9^Department of Pediatrics and Harvard Medical School, Harvard University, Boston, MA, United States

**Keywords:** adolescence, structural MRI, forgiveness, depression, anxiety, middle frontal gyrus, forgiveness and cortical volume

## Abstract

Dispositional forgiveness is positively associated with many facets of wellbeing and has protective implications against depression and anxiety in adolescents. However, little work has been done to examine neurobiological aspects of forgiveness as they relate to clinical symptoms. In order to better understand the neural mechanisms supporting the protective role of forgiveness in adolescents, the current study examined the middle frontal gyrus (MFG), which comprises the majority of the dorsolateral prefrontal cortex (DLPFC) and is associated with cognitive regulation, and its relationship to forgiveness and clinical symptoms in a sample of healthy adolescents. In this cross-sectional study (*n* = 64), larger MFG volume was significantly associated with higher self-reported dispositional forgiveness scores and lower levels of depressive and anxiety symptoms. Forgiveness mediated the relationship between MFG volume and both depressive and anxiety symptom levels. The mediating role of forgiveness in the relationship between MFG volume and clinical symptoms suggests that one way that cognitive regulation strategies supported by this brain region may improve adolescent mental health is *via* increasing a capacity for forgiveness. The present study highlights the relevance of forgiveness to neurobiology and their relevance to emotional health in adolescents. Future longitudinal studies should focus on the predictive quality of the relationship between forgiveness, brain volume and clinical symptoms and the effects of forgiveness interventions on these relationships.

## Introduction

Holding on to negative emotions can have significant adverse impacts on one’s mental wellbeing, whereas the ability to forgive can be protective against psychological distress (Worthington and Scherer, 2004). Forgiveness is comprised of cognitive, behavioral, motivational, or affective changes that promote positive social change with others or within oneself (Toussaint et al., 2016; Li et al., 2017), and is important during adolescent brain development, when clinical symptoms of anxiety and depression are emerging and escalating (Centers for Disease Control and Prevention [CDC], 2020b; World Health Organization [WHO], 2020). There is an approximately twofold increase in mood disorders between the ages of 13 and 18 (Child Mind Institute, 2017), and it is estimated that approximately 1.9 million adolescents in the United States have diagnosed depression and 4.4 million have diagnosed anxiety (Centers for Disease Control and Prevention [CDC], 2020a). As adolescents have enhanced susceptibility to the effects of stress, due in part to ongoing development of brain regions crucial for stress management (Tottenham and Galván, 2016), maladaptive coping and immature emotion regulation increase the risk of developing psychopathology in this age range (Compas et al., 2017).

Forgiveness has been shown to be protective against ailments ranging from depression to high blood pressure (Thompson et al., 2005; Worthington et al., 2007; Toussaint et al., 2016). In adolescent studies, positive relationships have been reported between forgiveness and several facets of wellbeing, such as self-acceptance, personal growth, self-assurance, and life satisfaction (Pareek et al., 2016; Barcaccia et al., 2020). Forgiveness is also negatively associated with anxiety (Flanagan et al., 2012) and depression in both healthy adolescents (Barcaccia et al., 2020), and in adolescents with psychiatric diagnoses (Dew et al., 2010). It is plausible that forgiveness could contribute to the development of emotional competence, while simultaneously reducing negative emotions.

Although the benefits of forgiveness on mental and physical health have been well documented, investigations of specific neural mechanisms underlying forgiveness are less well understood, with some neurobiological findings available in adult populations but virtually no existing studies in adolescents. In a recent meta-analysis (Fourie et al., 2020), interconnected components of forgiveness, e.g., cognitive control, were mediated by regions of the frontal lobe, including the dorsolateral prefrontal cortex (DLPFC), with five neuroimaging studies demonstrating relationships between forgiveness and larger DLPFC volume (Li et al., 2017) and greater DLPFC activation during tasks eliciting forgiveness (Brüne et al., 2013; Will et al., 2015; Ohtsubo et al., 2018). The cognitive control component of forgiveness was examined using a paradigm requiring participants to make a choice in response to an emotionally hurtful scenario; options were to forgive or to not forgive, i.e., continue blaming an imagined offender (Ricciardi et al., 2013). In the fMRI contrast comparing forgiving relative to unforgiving responses, significant activation was evident in left DLPFC, right inferior parietal lobule, and bilateral medial temporal gyrus. The results were interpreted as reflecting “reappraisal driven forgiveness,” given that choices to forgive required active reassessment of negative events as less negative (e.g., the transgression was “not too bad” or that transgressors “did not intend” harm).

Another cognitive strategy thought to support forgiveness is directed forgetting, which involves intentionally forgetting experiences, enabling individuals to move on more quickly and effectively from negative past events. While directed forgetting has positive impacts on wellbeing, directed forgetting is more difficult in individuals with depression (Joormann et al., 2009), due in part to negative attention bias, which has been shown to be present in adolescents with depression (Orchard et al., 2016). Successful reappraisal and directed forgetting require aspects of executive control known to rely on the functioning of DLPFC, including working memory, executive attention, and inhibition (Aguirre et al., 2017; Goldin et al., 2019), suggesting a major role for frontally mediated cognitive processes in forgiveness.

Although executive function and related brain areas are well-established to continue developing throughout adolescence (Casey et al., 2011; Luna et al., 2013), to date, there have been few studies of forgiveness and neurobiology that have included adolescents. In one such study, adolescents who reported experiencing chronic rejection, relative to adolescents reporting little or no rejection, exhibited increased brain activation in regions that included lateral PFC and dorsal striatum when they forgave unknown virtual participants who had excluded them during a virtual experimental game (Will et al., 2016). In another study of adolescents, Zhang et al. (2020) demonstrated that the relationships between forgiveness and depression were partially mediated by cognitive reappraisal, which relies upon inhibition and executive attention (Joormann and Gotlib, 2010; Messina et al., 2015), thereby also linking the forgiveness-depression relationship in adolescents to the DLPFC.

While there is ample evidence of DLPFC involvement using fMRI to examine forgiveness in adults, much of the literature further points to associations between forgiveness and middle frontal gyrus (MFG). The MFG, which comprises the majority of the DLPFC (Schilling et al., 2012), is a critical hub of frontal-limbic circuitry that mediates cognitive control and inhibition associated with adaptive coping and mental wellness (Merz et al., 2018) and that is involved with affective processing and depressive symptoms (Reynolds et al., 2014). Further, there are established links between MFG, development of cognitive control, inhibition, coping, and depression symptomatology during adolescence (Killgore et al., 2001; Blanton et al., 2004; Blakemore and Robbins, 2012; Flanagan et al., 2012; Pehlivanova et al., 2018). For instance, blunted MFG activation was associated with more externalizing symptoms (e.g., issues with behavioral inhibition) in adolescents (Heitzeg et al., 2014). Smaller MFG volume has been reported in adults during their first episode of depression (Han et al., 2014), whereas larger MFG volume was associated with resilience to major depressive disorder in individuals with high familial risk and a history of childhood maltreatment (Brosch et al., 2021). In addition, larger MFG volume was associated with better cognitive control, coping, and positive adjustment among adolescents who experienced adversity (Burt et al., 2016).

Accordingly, the objective of the current study was to elucidate relationships between MFG volume, forgiveness, and clinical measures (depressive and anxiety symptoms) in clinically healthy adolescents. It was hypothesized that (1) larger MFG volume would be associated with fewer depressive and anxiety symptoms, (2) more forgiveness would be associated with fewer depressive and anxiety symptoms, and (3) forgiveness would mediate relationships between larger MFG volume and fewer clinical symptoms. Identifying neural substrates associated with the potentially protective effects of forgiveness will be helpful for developing future studies aimed at ways to mitigate and/or prevent depression emerging during adolescence.

## Materials and Methods

### Participants and Procedure

Participants were 64 alcohol and substance naïve, healthy adolescents (13.86 ± 0.61 years of age, 81.3% white, and 51.6% female, see Table 1 for full demographics). The study protocol was approved by the Massachusetts General Brigham (MGB) Institutional Review Board. Adolescents and their parents provided assent and consent, respectively, prior to study participation. Participants were recruited locally using online social media platforms (e.g., Facebook), local flyers, and *via* partnership with Boston Children’s Hospital patient registries. Study procedures included magnetic resonance imaging (MRI), structured clinical interviews and clinical and self-report measurements. Adolescents were excluded based on MRI contraindications, serious physical health complications, history of head injury with loss of consciousness, presence of radiologic brain abnormalities, history of or current diagnosed psychiatric illness, and/or alcohol/substance use (more than a “few sips” of alcohol and/or any psychoactive substance use). Participants underwent urine screening prior to scanning to rule out substance use (Clarity Diagnostics Drugs of Abuse Panel, Boca Raton, FL, United States) and/or pregnancy (QuPID One-Step Pregnancy, Stanbio Laboratory, Inc., San Antonio, TX, United States). Adolescents were monetarily compensated for study participation.

**TABLE 1:** Demographics.

	*N* (%)
Biological sex (Female)	33 (51.6%)
**Racial identity**	
Black	1 (1.6%)
Asian	4 (6.3 %)
White	52 (81.3%)
Multi-racial*	6 (9.5%)
Not reported	1 (1.6%)
**Ethnicity**	
Hispanic	2 (3.1%)

*Participants who selected more than one racial identity were coded as “Multi-racial.”

### Clinical Measures

The Mini International Neuropsychiatric Interview for Children and Adolescents (MINI-KID) (Sheehan et al., 2010), a structured clinical interview, was used to determine psychiatric diagnoses based on the DSM-IV and to establish study eligibility. No participants met DSM criteria for any current depressive disorders.

Depressive symptoms were assessed using the Center for Epidemiologic Studies Depression Scale for Children (CES-DC) (Faulstich et al., 1986). The CES-DC consists of 20-items in which participants rate how frequently a statement is true over the past week using a 4-point Likert-type scale from 0 “Not at all” to 3 “A lot,” with total depressive symptom scores ranging from 0 to 60. Although scores greater than 15 may indicate clinical depression, the CES-DC is a screening tool rather than a structured clinical assessment based on DSM criteria for a diagnosed depressive disorder (Weissman et al., 1980). The CES-DC had an internal reliability of Cronbach’s α = 0.91 in this study sample.

Anxiety symptoms were assessed using the State-Trait Anxiety Inventory for Children (STAI-C; Spielberger et al., 1973). The STAI-C consists of two sub-scales, which measure trait anxiety and state anxiety. Only the STAI-C Trait subscale was used for the present analyses to assess dispositional anxiety rather than situational anxiety. The trait subscale includes 20 items in which participants rate how frequently a statement is true using a 3-point Likert-type scale from 1 “Hardly ever” to 3 “Often,” with total trait anxiety symptom scores ranging from 20 to 60. Scores were converted to t-scores based on normed data for sex and grade in school. The STAI-C Trait had an internal reliability of Cronbach’s α = 0.90 in this study sample.

The forgiveness measure was comprised of the 19-item Heartland Forgiveness Scale (Thompson et al., 2005) adapted for adolescents, and the 3-item Forgiveness-Short Form scale of the Brief Multidimensional Measure of Religiosity/Spirituality (Harris et al., 2008). Using a 4-point Likert scale of 1 = “Not at all like me” to 4 = “A lot like me,” participants rated themselves on four forgiveness dimensions including self-forgiveness (6 items, example: “Although I feel bad at first when I mess up, over time I can let it go.”); forgiveness of others (4 items, example: “With time, I am understanding of others for the mistakes they have made.”); situational forgiveness (6 items, example: “With time, I make peace with bad things in my life.”); and divine forgiveness (3 items, example: “I believe that God or a Higher Power has forgiven me for things I have done wrong.”). Nine items were negatively worded and reverse-scored for calculation of the total forgiveness score (minimum/maximum score range 19–76). The forgiveness measure had an internal reliability of Cronbach’s α = 0.78 in this study sample.

### Magnetic Resonance Imaging Acquisition and Processing

Imaging data were acquired using a Siemens TIM Trio 3.0 Tesla MRI system (Erlangen, Germany) with a 32-channel head coil. High resolution structural images were collected using a T1-weighted multi-echo magnetization prepared rapid acquisition gradient echo (ME-MPRAGE) 3D sequence in 4 echoes, using the following parameters: *TE* = 1.64/3.5/5.36/7.22 ms, *TR* = 2.1 s, *TI* = 1.1 s, *FA* = 12°, 176 slices, 1 9 1 9 1.3 mm voxel, acquisition time = 5 min.

FreeSurfer version 6.0 (semi-automated) reconstruction pipeline (Fischl et al., 2002; Fischl, 2012) was used to segment, label, and analyze T1-ME-MPRAGE images. To ensure high image quality, structural MRI data were visually inspected and manually edited. Volumetric files were visually inspected for accuracy of reconstruction, and no edits were necessary to those files. To control for head size, all neural regions were adjusted to each participant using estimated total intracranial volume (eTIV), a measure generated by FreeSurfer. To create a composite MFG volume, the four subregions were summed: right caudal middle frontal gyrus, right rostral middle frontal gyrus, left caudal middle frontal gyrus, and left rostral middle frontal gyrus (Kikinis et al., 2010). Manual edits were conducted and applied to the brainmask file, edits consisting of adjustments to pial surfaces to exclude dura matter (all files) and a minimal number of edits in which pial surfaces were adjusted to expand the white matter surface. Subsequently, volumetric files (aseg and subfield volumes) were inspected for accuracy of reconstruction, for which no edits were necessary.

### Statistical Analyses

Statistical analyses were conducted using SPSS 24.0 (SPSS, Chicago, IL, United States). Data were examined for outliers and coding errors, and for normality using skewness and kurtosis (skewness range: –0.377 to 1.297; kurtosis range: –0.548 to 2.006). Multiple linear regression analyses were performed to examine the associations between MFG volume and forgiveness, between MFG volume and clinical symptoms (STAI-C and CES-DC scores), and between forgiveness measures and clinical symptoms. All regression analyses included age and biological sex as covariates. No cases were outliers, i.e., three or more standard deviations from the mean, on any variable of interest. To evaluate whether forgiveness significantly mediated any relationships between MFG volume and clinical symptom levels, mediation was examined using the Baron and Kenny (1986) method. The size and statistical significance of the regression coefficient was examined for MFG volume in the models predicting depressive and anxiety symptoms when forgiveness was present vs. absent as a covariate. To confirm mediation findings, a bootstrap mediation approach was used, which produces ordinary least square estimates and 95% confidence intervals using 1000 bootstrap samples (Preacher and Hayes, 2008). Significance was measured at *p* ≤ 0.05. In the case of mediation analyses, Bonferroni corrections were made to account for multiple comparisons, with *p* ≤ 0.01 required to reach statistical significance.

## Results

In the current study, participants reported varying levels of depressive symptoms (*M* = 9.88 ± 8.74), trait anxiety (*M* = 38.45 ± 11.46) and forgiveness (*M* = 58.30 ± 7.51) (Table 2).

**TABLE 2:** Brain volume, clinical symptoms, forgiveness scores, and sex differences.

	Overall (*n* = 64)	Female (*n* = 33)	Male (*n* = 31)	Significance (*F, p*)
	Mean ± SD	Mean ± SD	Mean ± SD	
Total MFG	0.0350 ± 0.002	0.0346 ± 0.002	0.0354 ± 0.002	*F* = 1.68, *p* = 0.20
Depression	9.875 ± 8.740	9.27 ± 6.88	10.52 ± 10.44	*F* = 0.32, *p* = 0.57
Trait anxiety	38.453 ± 11.456	39.21 ± 8.98	37.64 ± 13.73	*F* = 0.30, *p* = 0.59
Total forgiveness	58.297 ± 7.514	57.51 ± 7.24	59.13 ± 7.83	*F* = 0.73, *p* = 0.40

*MFG, Middle Frontal Gyrus. Depression measured by Center for Epidemiological Studies Scale for Children. Trait Anxiety measured by the State-Trait Anxiety Inventory for Children. Female and Male denote sex assigned at birth, not gender identity.*

**FIGURE 1:**
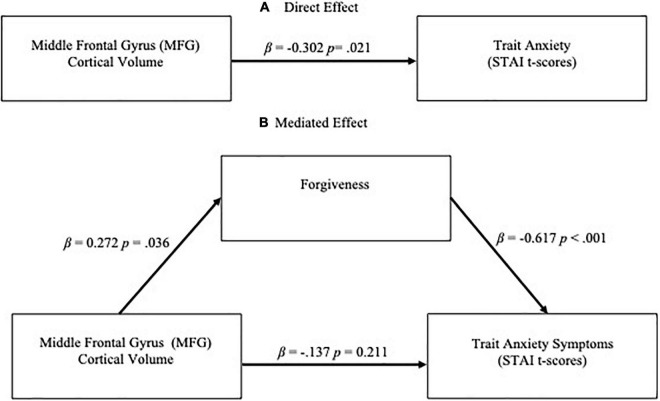
**(A)** Direct effect of middle frontal gyrus (MFG) cortical volume on trait anxiety (STAI *t*-scores) symptoms and **(B)** Mediation effect of forgiveness on relationship between MFG volume and trait anxiety symptoms. Significance was at the level of *p* < 0.01.

**FIGURE 2:**
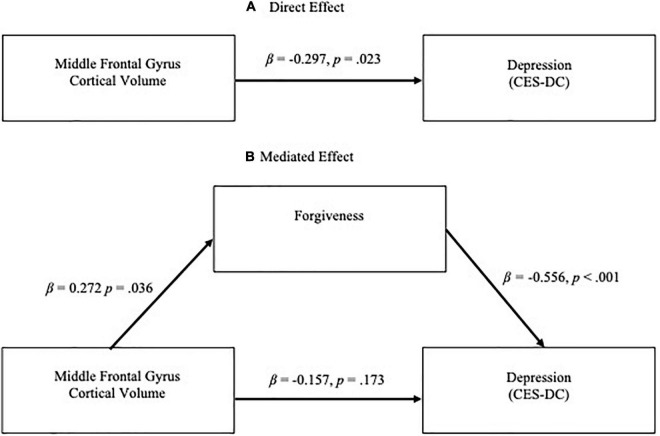
**(A)** Direct effect of middle frontal gyrus (MFG) cortical volume on depressive symptoms (CES-DC *t*-scores) and **(B)** Mediation effect of forgiveness on relationship between MFG volume and depressive symptoms. Significance was at the level of *p* < 0.01.

Both larger MFG volume and higher levels of forgiveness were significantly associated with fewer depressive and anxiety symptoms. In regression analyses controlling for age and sex, higher MFG volume was significantly associated with lower depressive symptoms [β = –0.297, *p* = 0.023; overall model *F*(3,60) = 2.208, *p* = 0.096, *R*^2^ = 0.099] and lower anxiety symptoms [β = –0.302 *p* = 0.021; overall model *F*(3,60) = 2.297, *p* = 0.087, *R*^2^ = 0.103] (Figures 1A, 2A). Similarly, in separate models, higher dispositional forgiveness was significantly associated with lower depressive symptoms [β = –0.556, *p* < 0.001; overall model *F*(3,60) = 9.227, *p* < 0.001, *R*^2^ = 0.316] and lower anxiety symptoms [β = –0.617, *p* < 0.001; overall model *F*(3,60) = 12.551, *p* < 0.001, *R*^2^ = 0.386]. Finally, MFG volume was significantly positively associated with forgiveness [β = 0.272, *p* = 0.036; overall model *F*(3,60) = 3.334, *p* = 0.036, *R*^2^ = 0.103].

Age and biological sex were not significant predictors in any of these models.

When forgiveness was added to the models analyzing the effect of MFG volume on clinical symptoms, controlling for age and biological sex, forgiveness was a significant predictor of depressive symptoms [β = –0.515 and *p* < 0.001; overall model *F*(4,59) = 7.499, *p* < 0.001, *R*^2^ = 0.337] while the effect of MFG volume became non-significant (β = –0.157, *p* = 0.173; Figure 2B). Likewise, in the regression model for anxiety symptoms, the added forgiveness variable was a significant predictor [β = –0.575 and *p* < 0.001; overall model *F*(4,59) = 9.819, *p* < 0.001, *R*^2^ = 0.400] while the effect of MFG volume became non-significant (β = –0.137, *p* = 0.211; Figure 1B). Consistent with the Baron and Kenny definition of mediation, dispositional forgiveness mediated the relationship between MFG volume and clinical symptoms. Bootstrap analysis verified these mediation results. The results remained significant after removing the aforementioned outlier.

## Discussion

In the present study, in clinically healthy adolescents, larger MFG volume was significantly associated with more forgiveness, as well as fewer symptoms of depression and anxiety, with forgiveness mediating the relationship between MFG volume and clinical symptoms. To date, this is the first investigation demonstrating a relationship between brain structure and forgiveness in adolescents, as well as the first study identifying a mediating role of forgiveness in the relationship between brain structure and clinical symptoms.

The current study findings are in line with previous studies showing links between MFG structure and depression and anxiety (Han et al., 2014; Molent et al., 2018; Wang et al., 2020). Particularly germane to the present study, the relevance of MFG volume to first episodes of depression (Han et al., 2014) suggests that this region may be a predisposing factor to the development and onset of some forms of psychopathology. The novel association between MFG volume and forgiveness in adolescents also parallels evidence of the more general DLPFC area, which has been associated with forgiveness in adults (Ricciardi et al., 2013; Li et al., 2017). The results of the current study likewise confirm previous reports that more forgiveness is related to fewer anxiety and depressive symptoms in adolescents (Flanagan et al., 2012; Barcaccia et al., 2020), as well as in adults (Webb et al., 2008), thereby adding evidence of mediation between variables to the existing literature.

The connection between MFG and clinical symptoms could be explained by numerous cognitive and emotional processes. Forgiveness relies on cognitive effort: the process of forgiving requires inhibiting feelings of anger and making a deliberate choice to let negative emotions go (Zhang et al., 2020). Cognitive reappraisal and directed (intentional) forgetting are cognitive processes that can aid in forgiveness, rely on executive function, and have been linked to MFG activation in fMRI research. Using fMRI, MFG activation was found to be related to cognitive reappraisal; when adults were coached to reappraise a perpetrator’s actions as more or less negative, participants reappraising actions as less negative showed increased MFG activation relative to a baseline condition (Grecucci et al., 2013b). Directed forgetting was also investigated in an fMRI study in which directed forgetting was compared with accidental forgetting of neutral and negative words (Yang et al., 2016). The results demonstrated increased MFG activation during directed forgetting of neutral words compared to incidental forgetting, and across the study it was more difficult for participants to forget negative words compared to neutral ones, emphasizing the cognitive effort required to extinguish negative content. The results of the present study, therefore, converge with prior fMRI findings, demonstrating a relationship between MFG structure and forgiveness, a process that can involve reappraisal and/or intentional forgetting (Noreen et al., 2014; Ho et al., 2020).

The role of cognitive regulation in forgiveness may also contribute to the observed relationship between MFG and forgiveness in the present study. The choice to let go of negative emotions—which is integral to forgiveness—can be achieved through deciding to view someone’s actions as less negative or choosing to not remember. The ability to reappraise actions as less negative could help abate depressive and anxiety symptoms by supporting forgiveness and the release of negative feelings, such as anger or sadness. Further, the current study suggests that the structure of MFG might be related to dispositional forgiveness, reinforcing the importance of the cognitive aspects of forgiveness in everyday functioning. Supporting this concept, MFG activation during a reappraisal task has been found to be positively correlated with the self-reported use of reappraisal strategies in daily life (Grecucci et al., 2013a), suggesting that MFG plays a role in reappraisal use outside of the laboratory. More broadly, forgiveness can be related to important evolutionary psychobiological mechanisms that undergo significant changes during adolescence; during this developmental period, motivational and emotional neurobiological systems help transition adolescents from competition to cooperation patterns (Giacolini et al., 2021), patterns than can be enhanced through forgiveness and that may reflect functionality of the MFG.

The current findings in this clinically healthy adolescent sample suggest that utilizing forgiveness could possibly provide a framework for early intervention strategies to prevent clinical symptoms from manifesting into diagnoseable conditions. Use of higher-level cognitive strategies to support forgiveness may facilitate downregulation of anger, which is an emotion known to enhance vulnerability for anxiety and depression symptoms (Daniel et al., 2009; Hirsch et al., 2012). In contrast, continued rumination in the absence of release—a release which could be facilitated by forgiveness—has been linked to greater depression and anxiety (McLaughlin et al., 2007; Ehring and Watkins, 2008). Thus, forgiveness could be an important strategy used to combat the development of potential psychiatric symptoms in adolescents.

Furthermore, the mediating role of forgiveness between the structure of a brain area known to support executive function and the manifestation of clinical symptoms suggests that existing therapies aimed at enhancing executive function (e.g., cognitive control or working memory training) (Koster et al., 2017; Jopling et al., 2020) and forgiveness interventions (Akhtar and Barlow, 2018) might be used in a synergistic way to maximize the effectiveness in treating adolescent depression and anxiety. Since the present study was conducted in a younger adolescent sample, the findings suggest that preventative efforts that integrate executive functioning skills and forgiveness could be potentially protective for the development of clinical disorders during later adolescence. Research demonstrates that failure to forgive is an indicator of poor mental health and encouraging forgiveness as part of an emotion-focused coping process during adolescence promotes healthier relationships and happiness (Rana et al., 2014). Elucidating relationships between forgiveness and neural processes can therefore help target future interventions to prevent anxiety and depressive symptoms.

There are several limitations that should be considered when interpreting the study findings. The current study did not apply any experimental manipulations of forgiveness, but rather utilized self-reported forgiveness, which has greater ecological validity, but can only be considered correlational in nature relative to brain volume and clinical symptoms. Thus, conclusion cannot be determined regarding directions of observed effects, i.e., whether forgiveness is a cause or effect of clinical symptoms or brain volume. While the focus on adolescents is a strength of the current study, it is worth noting that clinical symptoms and forgiveness during adolescence are developmental constructs, and therefore the cross-sectional nature of this analysis has inherent limitations regarding developmental changes in forgiveness, clinical symptoms and brain volume over time. Furthermore, the sample was predominantly homogeneous, with most participants identifying as Caucasian and representing little ethnic or racial diversity. Multicultural considerations are crucial for understanding mental health, and there is a complex relationship between race, racial discrimination and forgiveness (Powell et al., 2017). Thus, future studies should focus on populations known to be more vulnerable to mental health issues. Finally, while the mediation analysis employed, which was confirmed by bootstrapping, enhances the rigor of this statistical approach (Agler and De Boeck, 2017), there is clear need for additional research in this area using more sophisticated analysis methods.

Future studies employing longitudinal designs should focus on the predictive quality of the relationship between forgiveness, brain volume and clinical symptoms, and the effects of forgiveness interventions on these relationships. Further investigations could also focus on different facets of executive functioning to potentially isolate cognitive elements of forgiveness, especially those that are rapidly maturing during adolescence. In the current era, marked by tension and divisiveness, as well as mounting depression and anxiety associated with the global COVID pandemic, forgiveness is critical to help build a more united future; this study highlights the relevance and promise of forgiveness to positively interact with neurobiology and promote emotional health, particularly in youth.

## Data Availability Statement

The raw data supporting the conclusions of this article will be made available by the authors, without undue reservation.

## Ethics Statement

The studies involving human participants were reviewed and approved by Massachusetts General Brigham Institutional Review Board. Written informed consent to participate in this study was provided by the participants’ legal guardian/next of kin.

## Author Contributions

All authors listed have made a substantial, direct, and intellectual contribution to the work, and approved it for publication.

## Conflict of Interest

The authors declare that the research was conducted in the absence of any commercial or financial relationships that could be construed as a potential conflict of interest.

## Publisher’s Note

All claims expressed in this article are solely those of the authors and do not necessarily represent those of their affiliated organizations, or those of the publisher, the editors and the reviewers. Any product that may be evaluated in this article, or claim that may be made by its manufacturer, is not guaranteed or endorsed by the publisher.
